# COVID-19 and mental health in children and adolescents: a diagnostic panel to map psycho-social consequences in the pandemic context

**DOI:** 10.1007/s44192-021-00002-x

**Published:** 2021-10-25

**Authors:** Menno Baumann

**Affiliations:** Fliedner-Fachhochschule Düsseldorf, Düsseldorf, Nordrhein-Westfalen Germany

## Abstract

Since the beginning of the COVID-19 pandemic, much research has been done on the psycho-social consequences, especially for children, adolescents and families. In the long run, there is a large set of quantitative data available. However, these still seem to be not well understood. Theoretical classifications of the evidence also diagnostic tools still seem to be open. This paper elaborates a possible systematisation based on theoretical models of systemic self-organisation theories. This leads to a model for a comprehensive psycho-social child-in-environment diagnostic to map potential problem areas. Such a theoretical framing should enable both: a deeper understanding of the impact of pandemics on young people and hypotheses for intervention strategies in the context of pandemic management as well as in the context of diagnostic-systemic interventions in psycho-social working settings. In the coming months and years, it will be essential to be able to understand and describe psychosocial disabilities that have developed in the context of the pandemic in a differentiated way in order to establish targeted interventions.

## Instruction

After the novel coronavirus SARS-CoV-2 spread very rapidly across more or less the whole globe at the beginning of 2020, measures were taken in almost all countries of the world to contain the outbreak. Because it was still an unknown and essentially unresearched virus, only the wellknown non-pharmaceuticel interventions [1] were available to fight the pandemic. It was rapidly discussed that measures such as social distancing, school closures, and social contact limitations would not only have an impact on the pandemic process, but also on an individual’s psycho-social health.

Even before the COVID-19 pandemic, it was known that health threats also have a psychological impact [1]. There are also effects and collateral damages of non-pharmaceutical interventions (NPI) proven by scientific studies, e.g., from the large Ebola outbreak in West Africa in 2013–2014 [2, 3] or the outbreak of H1N1 (swine flu) in 2009 [4].

However, that the COVID-19 pandemic would have such a strong impact on people's daily lives in much of the world for more than a year and a half caught most parts of that world completely unprepared. In Central Europe in particular, discussions flared up as to whether COVID-19 had any more serious health consequences than the restrictions on social life and widespread isolation. These discussions mainly concerned children and adolescents, who seemed to suffer particularly severely from the restrictions. Just as quickly as this discussion made it into the headlines of major newspapers, however, it also seemed to end when, in the context of adult vaccination advances, increasingly relaxed containment measures were implemented.

Both for the reflection of the pandemic, the question of what can be learned for future scenarios comparable to the pandemic, and for the post-pandemic phase, it seemed important to the author to review the state of knowledge of psychological and social science research and to theoretically transfer it into models that enable professionals to diagnostically evaluate psycho-social risks of children and adolescents and to develop support measures accordingly. Especially now, it seems important not to persist in headline-grabbing generalizations and empty wordsmithing (such as the completely unscientific formulation of the “long lockdown syndrome” in social networks). Instead, a theory-based review of the psychological evidence is needed to draw conclusions for the support of young people, both to be able to support young people appropriately in their normal development in the future and to target those who have been really seriously affected by the pandemic in their social and family environment and/or in their mental health.

This perspective article aims to synthesize the rapidly growing amount of research on the impact of the pandemic on child, adolescent and family mental health through a mapping review and to place it within theoretical models. The method of a mapping review was chosen in order to visualize the state-of-the-art of research for educational and therapeutic practice and thus to present and make it available within the framework of a diagnostic panel. This may be the baseline for pedagogical, psychological and medical practitioners to properly sequence problems that arose, to understand them in a child-in-environment diagnostic, and to derive strategies of support from them. For further dynamic phases of this pandemic, for the hopefully soon reached time of the post-pandemic as well as preparatory also for perhaps future pandemics and epidemics.

## Methods

**Fig. 1:**
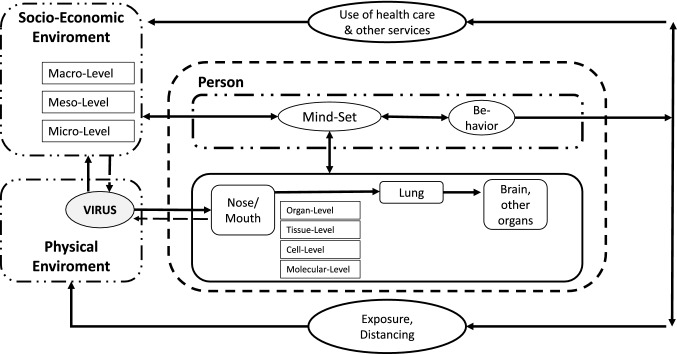
Systemic bio-psycho-social model of individual being in confrontation with a pandemic (modificated according [5])

**Fig. 2:**
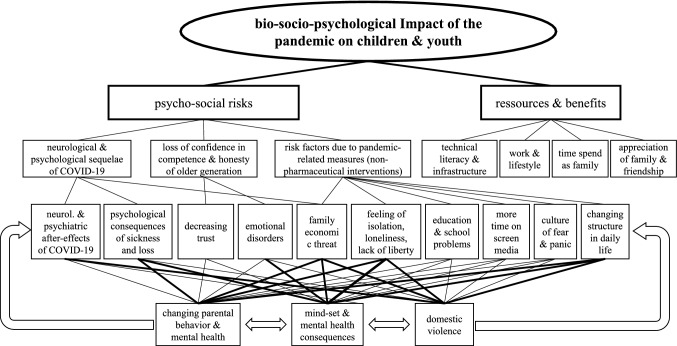
Structural analyses of psychosocial burdens of the COVID-19 pandemic

In order to provide guidance to professionals working in the psycho-social domain for the assessment of mental health and psychological impact of the pandemic in children and adolescents, a theory- and model-based mapping review was conducted. In the first step, aspects and questions were elaborated on the basis of the self-organization theory and its bio-psycho-social conception of human beings presented in medicine (Fig. 1, [5, 6]) as well as the theoretical systematizations of psychological pandemic impact by Taylor [1] and Fitzgerald et al. [7] (Fig. 2). In the second step, the psychological evidence regarding the COVID-19 pandemic was then extracted. The databases pubmed, pubpsych, and google scholar were used to search for studies on the specific questions to determine which aspects could be shown to be scientifically evident. The aim was not to include all available studies (systematic review), but to support or refute the hypotheses within the framework of the theoretical models. The steps—matching the article titles, analyzing the abstract, reading the entire paper, inclusion in the mapping review—were carried out until the research question appeared to be adequately covered by the included studies. In the third step, the results were visualized in the model of the bio-psycho-social conception of human beings (mapping dashboard), in order to prepare them for practical work with children and adolescents under stress, and supplemented by the existing models of family violence and pandemic conditions [8] and the factors influencing parental parenting behavior [9], in order to present these relevant individual questions in a more differentiated way.

### Theoretical model I: self-organisation in psychology

Self-organisation theories have a decades-long tradition in the study of life. They have also gained increasing influence in medicine and psychology [5, 6, 10]. The basis for this is the assumption that complex systems constantly maintain themselves (autopoiesis), regulate and reproduce themselves by means of various circular processes (feedback loops) built up hierarchically through top-down and buttom-up processes [11]. These processes, which are also effective in the interaction with the environment, must be seen as non-linear processes [10].

Fundamental to this is the cycle of structure and activity. Each system state is the result of the structure of a system, simultaneously acting back on it and changing it so that a new state of the structure emerges.

This process applies to intra-organismic processes in the context of the activity of the central organisational systems of the organism, e.g. the organs, the immune system, the hormone system as well as the neurotransmitter systems of the brain [11, 12]. In the same way, these system dynamics also apply to mental processes and the organisation of the mind [10] and at least also of the organism as a whole.

Circular dynamics are also a basic principle in the interaction with the various levels of the environment. The environment never has a linear-causal effect on a person. How an organism can integrate its environment into the establishment of a new equilibrium depends essentially on the state of the organism itself. In the context of the bio-psycho-social conception of human beings [5, 6], the environment can be divided into several levels. Following Bronfenbrenner [13], a distinction is made in systems theories between the micro-systems, which describe the immediate interactions of the individual. At the level of the meso-system, those dynamics are grasped which take place between the systems with which the individual interacts directly. For example, the communication between the parents influences the interaction qualities between the child and the respective parent. On the macro-system level, on the other hand, processes and social structures are described that run largely independently of the individual (e.g. value systems), but which exert a great influence on individual development and the respective interactions at the micro-system level.

In the same way, the physical environment does not affect the individual mechanically. For example, neuroscientific studies have shown that the consequences of brain damage caused mechanically from the outside (e.g. by an accident) are clearly dependent on inner-organismic processes, including the hormonal system and the psychological processing of the trauma [14]. Rather, the physical environment provides the context for social systems and is actively appropriated by the individual.

Tretter, Wolkenhauer, Meyer-Hermann, Dietrich, Green, Marcum and Weckwerth [5] have worked out such a systemic framework in the context of the COVID-19 pandemic (see Fig. 1).

### Theoretical model II: the bio-psycho-social impact of pandemics on children and adolescents

The psychological consequences of the pandemic for children, adolescents and families cannot be considered in such a model simply as linear-causal factors. Here, as well, a wide variety of conditioning factors can be seen. However, the core of the consideration must be that the factors that have an impact on the bio-psycho-social organism on the one hand act on a complex system whose individual structure essentially determines a pandemic's impact. On the other hand, the individual factors also influence each other. Whether a certain influencing factor has an effect even influences or compensates for the respective effects of other factors.

For example, the closure of schools on the one hand can have a negative effect on a person due to a lack of daily structure, difficulty in reconciling family and work and problematic [15–17] acquisition of educational content. At the same time, however, it can also lead to a reduction in anxiety, fewer infections in the child's environment [18, 19] or even an increase in learning because disruptive factors, e.g. an unfavourable class structure, are eliminated.

Similarly, a single factor may be a risk factor in one case, but a resource in another. For example, one family in lockdown may benefit from more individual time for the child due to a more conscious allocation of time [20]. For another family, on the other hand, the lockdown can mean severe poverty, be very stressful due to cramped living conditions and lead to an increase in conflicts, violence and psychological stress.

This points to the fact that a review of the scientific evidence on psychosocial stressors needs to be embedded in a theoretical model in order to be understood in its dynamics. The Australian working group of Fitzgerald, Nunn and Isaacs [7] have put the current state of scientific evidence of the consequences of pandemic (measures) into a hierarchical structure of headings, which forms the basis of the model discussed here. They distinguish between costs and benefits, and in the case of costs between psychological and social consequences. Furthermore, a distinction must be made between the consequences of a COVID-19 infection on the psyche, the experience of how the adult generation faces failure and the social and psychological consequences of the non-pharmaceutical pandemic measures.

In a theoretical model based on hierarchically structured but circularly organized impact factors, the evidence of the psycho-social consequences of the pandemic can be summarized as shown in Fig. 2.

## Results

From these preliminary theoretical considerations, the empirical evidence of each factor was then extracted and transferred to the model (Fig. 1) to expose the visualization of the mapping review as a result of the study.

### Ressources and benefits

The first path to follow asks about resources and benefits of the changed pandemic situation. The technical infrastructure and the technical literacy of many people has significantly improved [7, 21]. The lifestyle and organisation of work has also been changed in favour of the children in some families that had the appropriate economic and educational background at the beginning of the pandemic [15, 21]. Thus, time spent as a family can also be considered by many young people as quality time that strengthens the attachment to parents, regardless of the young people’s age [20, 22, 23]. The pandemic also brought new awareness to the attitude and appreciation of family and friendships. Of course, these factors in no way mitigate the risks and negative effects on many young people, but there is a high scientific evidence that the pandemic crisis was also able to trigger positive effects in families that entered the crisis with little stress. It is important to note that the findings of the research on the psycho-social consequences of lockdowns showed that most families had the necessary resources to cope with a clearly structured lockdown without major problems [23–28].

### Sequelae of an infection with COVID-19

Another pathway that needs to be considered as a risk factor in the mapping of psycho-social consequences is the direct sequelae of infection and illness. SARS-CoV-2 infection can have long-lasting and severe consequences. These include severe organ damage and the so-called longCOVID, which can also affect children [29–32]. However, there is now also a large scientific evidence that infection can also result in a number of neurological and psychiatric disorders, with up to 34% probability of occurrence within 6 months [31–38]. This constitutes both a direct risk for young people, but of course also an indirect one if severe health damage or mental illness affects the parents.

### Loss of confidence in competence and honesty of older generation

An issue that is completely underexposed in public discussion is the declining trust and disenchantment due to pandemic policies and adult behaviour and communication. Young people recognise contradictions, dishonesty and lack of decisions and their psychological well-being is affected [7, 39, 40]. A German survey with young people showed that juvenile suffered from the lack of opportunities for participation and were disappointed by the reduction to their role as students [21]. Developmental researchers in the USA also explicitly warn against the harm caused by the narrative of the “lost generation” as an adult assessment [41–43].

### Consequences of the pandemic measures

And finally, of course, there are the psycho-social consequential harms caused by the pandemic control measures (non-pharmaceutical interventions). Social distancing, school closures, restrictions on social life and the threat to the economic existence of families in the context of so-called lockdowns have considerable consequences for the development of young people. One of the greatest dangers at this point is when families are thrown into poverty by the pandemic [8, 22, 44–48]. But emotional problems, especially fear, isolation and boredom, are also a burden [1, 21, 22, 49–52]. Distance learning and school closures also lead to educational inequity and less learning success [28, 48, 53, 54]. More time in front of screen media can also have a problematic impact both directly, but especially violence against children on the internet seems to have increased [55–57]. Especially for younger children, changes in daily structure seems to have a high impact as well [8, 16, 58, 59], while adolescents also perceive the omnipresent “culture of panic” [7] as a burden.

## Proposal of a diagnostic model for contextualized mapping review of risk factors

All these risk factors are well known and can be considered scientifically evident. But a reduced view of statistical probabilities alone cannot explain why individual development paths vary so much. To understand the evident, they need to be integrated into qualitative models within which interrelationships, dynamics and circular processes can become clear.

Initially, it must be noted that all these factors will change the balance of complex self-organised and circular systems. First, this is always done on the basis of the historically grown structures of the system that a factor encounters—the individual, the family, the relationships. Secondly, the individual factors are reciprocally dependent on each other. No single factor stands on its own. For example, in the case of educational policy, the closure of schools can lead to a feeling of social isolation and cause problems for learning. But school closures limit potential illnesses and their consequences and reduce individual quarantine measures, which in turn can be highly problematic. At the same time, however, it could also activate resources, e.g. in the parent–child attachment or in technical skills. Opening schools in the same pandemic situation, on the other hand, can offer both relief, social contacts and daily structure. At the same time, however, it can reduce the young person's trust in the adult experience, which imposes harsh restrictions in private and forces many children into a closed space at school. Also, increased infections can lead to increased psychological and physical problems, up to long-term consequences or even deaths in families [18, 19, 60, 61]. The psychosocial consequences of mass quarantines [45, 62–67], as they occur with high incidence and attendance classes, would also have to be included in a consideration. It is therefore not possible to simply say in a linear-causal way that schools in face-to-face education protect children from psychological problems. Within dynamic systems, the opposite can also be the case in individual contexts. And this applies to every single factor.

So already at the level of risk and resource factors, a linear approach X → Y is not sufficient, but complex dynamic equilibria have to be analysed.

In the final analysis, all of these factors are mediated by three process variables that cannot be located in a linear-causal way, but are interrelated in a highly complex structure of self-organising dynamics: changes in parental behaviour [21, 59, 68, 69], changes in the social-emotional experience (mind-set) and behaviour of young people [1, 28, 51, 52] and changes in the conflict dynamics of families, up to and including domestic violence [8, 70–73]. These three mechanisms of action (operators) are in turn circularly integrated into a general picture, both a consequence of the pandemic measures and a condition for their psycho-social consequences. Domestic violence for example is not caused by non-pharmaceutical interventions or a lockdown in the narrow sense. But a vulnerable system can be so disrupted by the measures that it arises. Where family violence is or becomes part of the system, this in turn has a multiplier effect on all other negative effects and hence maintains or reinforces itself.

The mapping review developed here provides a structure to conceptualize the empirically documented factors of pandemic impact in a way that can be used to derive action strategies to support youth.

For this purpose, the model of a bio-psycho-social view of the individual in the pandemic outlined above is first used to illustrate the dynamics of the pandemic in terms of a child-in-environment analysis. In the second step, the influence of the pandemic on parents is reflected on the basis of the model “determinants of parenting behavior” according to Abidin [9]. Finally, in the last step, a “path model of domestic violence among non-pharmaceutical interventions” one area that needs to be looked at more closely is the behavior of a child’s key caregivers at the micro-level of the social environment [8] is used to propose a way of analyzing family conflicts.

### The mind-set and the psychic system

**Fig. 3:**
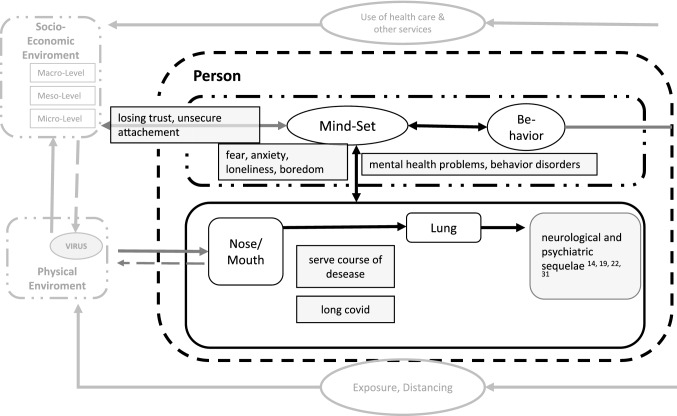
In person-factors (modificated according [5])

In the model of the bio-psycho-social view of human being described at the introduction, the psyche is a system embedded in the organism as well as in the social and physical environment. The risk factors just described act on the different levels and thus change the flow equilibrium. First of all, there is the effect on the organism (compare Fig. 3). Severe courses of disease, longCOVID and psychological and neurological sequelae have an effect [29–31, 34–38, 74, 75]. But in addition to the organic effects of potential viral diseases, the pandemic also has a psychological impact independent of the question of whether a child itself was infected. Emotions such as fear, anxiety, loneliness as well as boredom are reinforced by various pandemic measures [1, 21, 22, 49, 51, 52, 76, 77]. This has also led to an increased incidence of mental illness in children and adolescents during the Corona period [24, 52]. In addition, the young person's perception of the pandemic as a whole is influenced by the behaviour and communication of adults and political decisions [7, 21, 39, 42].

All these aspects can be influenced—depending on the mind-set and perception of the situation—both by containment measures and by measures not taken.

### The socio-economic environment

The young are embedded in their social environment. This in turn is massively changed by the pandemic conditions. At the level of immediate relationships (micro-level), this primarily affects the family and the closest friendships. Of course, these most important attachment figures are also exposed to considerable psychological risk and their behaviour towards the child changes [22, 39, 68, 78]. Changes in the common daily structure [16, 58, 59, 78, 79] and in conflict behaviour (up to violence) can become the new system conditions in this context [8, 68, 71, 72, 80].

The meso-level includes the relationships, communications, and activities of the systems immediately around the child. Here, processes of anxiety, isolation, and loneliness can also emerge, depending on the degree of actual isolation and perception [69, 70]. Role relations, for example, between parents, can also shift [3, 8, 16], which can be both a resource (in the case of more equality and role distribution) and a threat (in the case of a patriarchal role model). Violent relationship patterns can also be manifested under conditions of narrowness and lack of escape options at the parental level [8, 39, 81].

However, the social and economic changes in the environment, which extend far beyond the children's immediate sphere of influence, can also have a major impact on the psycho-social condition of the individual. Probably one of the greatest risk factors is when the family experiences economic and existential hardship [1, 7, 44, 46, 53, 73]. The processes of educational injustice exacerbated by distance learning [48, 53, 54] also build up high pressure on families. And finally, communication about the pandemic at the societal level also affects the psychological experience of the individual. Thus, both a “culture of panic” [7] can be a risk factor, as well as a growing divide between conspiracy theorists and people who support the measures [1, 49].

**Fig. 4:**
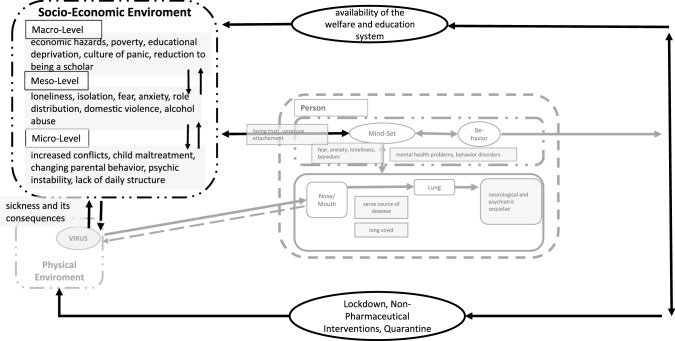
Impact-factors of socio-economic environment (modificated according [5])

When considering the environmental factors that may affect the child or adolescent as a result of the pandemic (Fig. 4), it is of course important to consider if there have been infections in the local environment and if these have left consequences that have a psychological impact on the health of the caregivers or other important human [32, 33, 35, 36, 38, 82, 83].

### The physical environment

In addition to being socially embedded in the environment, individuals are also embedded in their physical environment. Of course, SARS-CoV-2 is also part of this physical environment.

Conditions of the physical environment that influence the impact of psycho-social exposure are, for example, housing conditions. Cramped living conditions, use of poorly ventilated stairwells or elevators, lack of opportunities to exercise in one’s own backyard all complicate infection control and also increase the severity of harm from lockdown, curfews, or isolation in quarantine [26, 53, 62, 63, 65, 66, 84, 85].

Another factor is infection protection in places the individual may not have the ability to avoid [19]. Public transportation, schools, supermarkets—these are all places that, if poorly secured, can cause fear, insecurity, opposition, and, of course, real infections.

A third important factor in the “physical environment” is the increased insecurity for children and adolescents online [57]. As it stands today, young people were more likely to be victims of assault than before the Corona crisis.

As a subset of the physical environment, it is also necessary to consider the measures actually implemented, which can vary widely in their impact [22, 26, 27, 45, 73]. In this context, the lack of support systems and closed educational institutions have been found to be particularly significant [3, 8, 53]. However, the interaction of different choices must be considered separately. For example, opening up social life as much as possible may well have negative psychological consequences if a large number of quarantine measures have to be imposed due to high incidence and contact density [62–64, 67]. The consequences of decisions therefore affect not only the infection process, but also the socioeconomic environment.

**Fig. 5:**
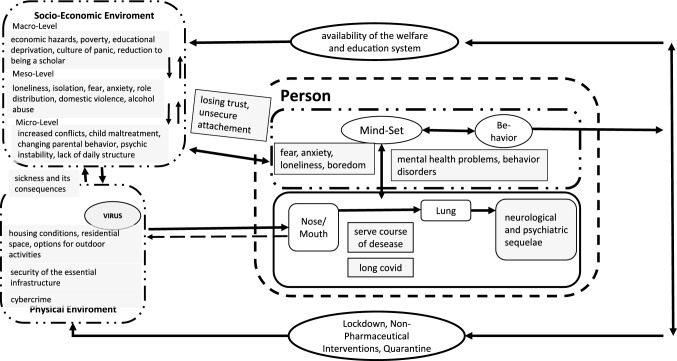
Base model for mapping psycho-social impact in a child-in-environment diagnostic (modificated according [5])

Thus, the overall picture reveals a complex analyzing model for mapping the psycho-social sequelae in the context of a child-in-environment diagnosis (groundes on Fig. 5). In addition to this analysis, the dynamics of the other two mechanisms of action, the change in parental behavior and the change in conflict patterns, must also be examined in detail.

### Changes in the behavior and conflict dynamics of the caregivers

One area that needs to be looked at more closely is the behavior of a child’s key caregivers at the micro-level of the social environment.

**Fig. 6:**
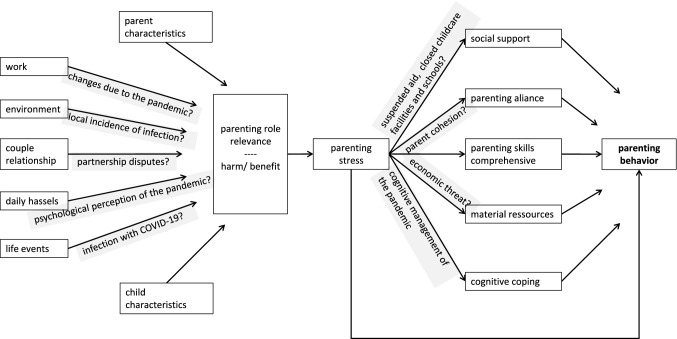
Determinants of parenting behaviour and pandemic effects (modificated according [9])

Parenting behavior depends on different variables, which Abidin [9] has summarized in a path model (see Fig. 6). Again, pandemic impacts can be described and differentiated on the various determinants. For example, the pandemic can both increase risk potential and make resource activation more difficult. For the question of child-in-environment diagnostics and pedagogical-therapeutic interventions, it is important to differentiate impacts of the pandemic on the psycho-social condition of children and adolescents in such a way that the effect mediated by the changed behavior of the caregivers becomes representable (Fig. 6).

Finally, of course, it is very similar factors that define parental behavior toward the child. The character traits of parents and child enter into the interaction regardless of the pandemic. In contrast, other areas such as work life, the economic situation [15, 17, 46] and related fears [1, 49], the change in the environment due to measures [1] but also due to infections (up to one’s own illness) [36, 60, 61], one’s own perception and assessment of the pandemic, and relationships at the parental level and in the friend network [69, 70] are strongly influenced by the pandemic. The relationship and/or attachment between parent and child pre-Corona naturally moderates the strength of the influence of each aspect, but is in turn circularly influenced by them for the future. The specific behavior of parents is thus influenced by a combination of how the pandemic affects them and what resources they can activate under these conditions (social distancing, school and daycare closures).

**Fig. 7:**
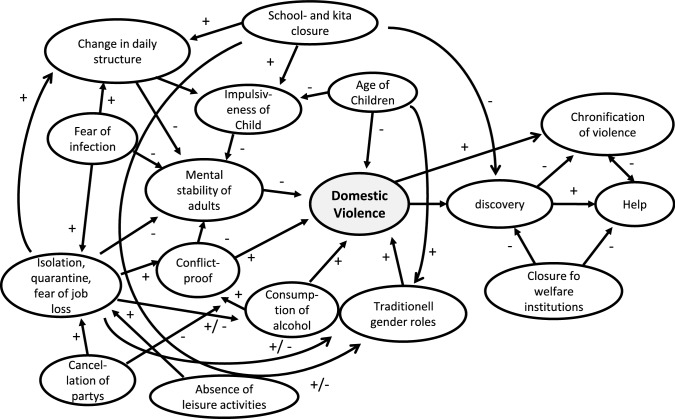
Path model of domestic violence and the effect of non-pharmaceutical interventions (modificated according [8])

Beyond the scope of parental custody behaviors, family conflict and domestic violence during restrictive pandemic restrictions such as lockdown, quarantine, contact restrictions, social distancing, and curfews appear to be a particular risk factor for children that needs to be considered more closely in terms of its dynamics [3, 73, 86]. Especially with regard to conflict tensions, which seem to have increased significantly in the phases of severe restriction [21, 52], increased alcohol consumption [81], shifting daily structures, and male and female roles [3, 16, 59, 79] are important points of observation. In some families, such processes may have increased to the point of family violence [8, 56, 71–73, 80]. Accordingly, a systemic diagnosis of the negative consequences of the pandemic for the development of children and adolescents needs a family dynamic analysis of the potential for conflict and violence, as shown in Fig. 7 [8].

## Discussion

This paper has shown how complex the influences of a pandemic situation on children, adolescents and families are. There are few linear effects. Rather, psychological development as well as the family as a system are in a state of self-organized steady state. From such a complex point of view, there is a need for differentiated models and instruments for pedagogical–psychological diagnostics. Only with such a basis interventions in the acute phase of a pandemic as well as in its aftermath can be targeted to the needs and problem areas of the individual young person in his or her family situation.

In addition to providing a deeper understanding of the psychosocial consequences of the COVID-19 pandemic for children and adolescents up to this point, the study also offers practical implications for the educational and, where appropriate, therapeutic guidance of young people. The diagnostic dashboard developed as part of the mapping review provides a basis from which specific questionnaires and items can be developed to provide a rapid assessment of which of the named risk factors are or have been effective in a young person, and which resources have been or can be activated. It seems significant to offer support to the professionals here, so that after a long phase of warning, also a phase of active intervention is possible.

It seems to be of elementary importance to focus now on empowering and restabilizing young people in their emotional experience. To do this, they need educational services, counseling (especially for parents), empowerment-oriented support, time and, in difficult cases, rapid psychotherapy or family therapy help. The focus must not be placed on resolving deficits, such as poor grades, but rather on skills and social needs.

In addition, it is important to address the important components in counseling parents and families. For example, that an increase in alcohol consumption is a significant risk factor, that especially anxiety and stress (but also anger) of parents can become difficult for children and therefore must be reduced through the use of counseling, how important a daily structure is, especially in quarantine or lockdown—all these are factors about which families need a lot of information and targeted offers.

Politically, risk factors such as the threat of poverty or poverty caused by the pandemic, precarious employment or poor childcare options can also be given additional support in order to get through such a time better in future crisis situations. Testing capacity and hygiene efforts need to be particularly focused on educational settings to quickly offer children and families a return to normalcy. Considerable potential is also still evident in the area of communication. Information services should also be aimed specifically at adolescents and at children. They should also be involved in decisions in a manner appropriate to their age and, above all, should under no circumstances be given the impression that they are simply being forgotten (e.g., in the case of contact restrictions, vaccination quotas, etc.).

## Limitations

The model is based on a mapping review, not a systematic review that included all available sources. There was no negative selection in the sense that studies were excluded because their results did not match other results (selection bias). However, it cannot be excluded that there are also important studies that were not included. Nevertheless, the search was much more extensive than in a rapid review, which can be regarded as an appropriate alternative in view of the high time pressure and the high relevance of the topic.

The search took place in the period February to June 2021. During this time, many new research papers were published, some of them still as preprints. In the period after June 2021, many more studies have undoubtedly been published, which could further differentiate the picture. It also cannot be ruled out that individual preprint publications were judged to be inadequate and limited in their informative value during a peer review process. When the article was completed, the sources were checked again, and no study has been withdrawn since the research was completed.

Also, the research was based on the theoretical models from self-organization theory [5] and from the elaborations of Taylor [1] and Fitzgerald et al. [7]. Admittedly, the abundance of data processed in this paper must be considered extremely extensive compared to other perspective articles for a statement on the scientific evidence of psychosocial risks for children and adolescents at the current time. Nevertheless, it cannot be ruled out that a different theoretical foundation would have focused on other studies and some articles central to this paper might not have been considered.

## Future research requirements

From this research, in addition to the practical implications for working with children and adolescents, several other research perspectives emerge. The model does not contain any type of weighting of the single factors, since the included studies were carried out methodologically very differently. Only the direction of certain influences are pointed out. An empirical overall consideration of the model may show which of the factors found have particular relevance, which of the factors have a high comorbidity, and which resources may also have a particularly strengthening effect on resilience with regard to certain risks.

Likewise, further research can be conducted directly with the dashboard presented. This can provide deeper insights into the psychosocial situation of young people, going beyond the mere question of whether or not stresses are present.

An important step would also be to differentiate for interventions in future crises: what risks can be mitigated through policy action? For which aspects do we need counseling and therapy services that are quickly available in crises? What can families, neighborhoods and districts do to support each other in an exceptional situation such as a lockdown and to strengthen resilience? Such differentiation will help to cope better and faster with global events like a pandemic in the future, as a psycho-socially resilient population will also implement necessary crisis interventions and NPI’s more easily and consistently.

## Data Availability

Not applicable.
